# Diagnosis and Treatment of Soft Tissue Tumours: The Dutch Nationwide-Accepted Consensus

**DOI:** 10.1080/13577149877957

**Published:** 1998-12

**Authors:** A. N. Van Geel, J. A. M. Van Unnik, R. B. Keus

**Affiliations:** 1 Surgical Oncology Rotterdam Cancer Institute/Daniel den Hoed Cancer Center Rotterdam The Netherlands; 2 Pathology Bosch en Duin Amsterdam The Netherlands; 3 Radiotherapy Netherlands Cancer Institute/Antoni van Leeuwenhoekhuis Amsterdam The Netherlands


*Sarcoma* (1998) **2**, 183–191

ORIGINAL ARTICLE

### Diagnosis and treatment of soft tissue tumours: the Dutch nationwide-accepted consensus

A. N. VAN GEEL,[1] J. A. M. VAN UNNIK[2] & R. B. KEUS[3]

On behalf of the Dutch Cooperative Group for Soft Tissue Tumours

[1]*Surgical Oncology, Rotterdam Cancer Institute/Daniel den Hoed Cancer Center, Rotterdam &* [2]*Pathology, Bosch en Duin &* [3]*Radiotherapy, Netherlands Cancer Institute/Antoni van Leeuwenhoekhuis, Amsterdam, The Netherlands*

### Introduction

A reasonable number of soft tissue sarcomas are found by chance. The reason is that general surgeons are not familiar with these cancers and sometimes do not realize that an 'atypical lipoma' or a 'hernia of the muscle' could be a soft tissue sarcoma. Whether these 'whoops' operations badly influence the prognosis is uncertain, but it is certain that definitive surgery can be very complex with increased morbidity and frequently needed adjuvant radiotherapy.

With this knowledge in mind, the Dutch Cooperative Group for Soft Tissue Tumours, founded in 1991, recognized this important issue. The first activity involved organizing a nation-based consensus meeting. To achieve this goal all cancer centres (2), universities (6) and comprehensive cancer centres (9) were asked to send representative participants to prepare a draft report. On 29th October 1993, after two-and-a-half-years and nine meetings, the text was open for discussion. Thereafter the definitive text was sent to all surgeons and to specialists involved in soft tissue sarcoma treatment (ISBN 90-6910-164-5). A shorter version was published in the national general medical journal (*Ned.Tijdsch.Geneesk* 1995; 139: 833–7).

The original text is now published in this journal. The intention is that this text, based on 28 main questions, can be helpful to those people who are also preparing a regional or national consensus. Alternatively this text could be encouraging those doctors with a special interest in soft tissue sarcoma to prepare such a consensus.

### Basic questions

1: What order in diagnostics should be followed in patients presenting with a suspicious soft tissue tumour?

2: What conditions can be made for the preparation of pathology specimens and how to report the results?

3: What are the therapeutic possibilities of surgery, radiotherapy and chemotherapy in the treatment of primary soft tissue sarcoma?

### Definition and classification

### *1. Soft tissue sarcomas form a heterogeneous group of uncommon tumours with histopathological aspects of connective, muscle, fatty tissue or peripheral nerve tissue*

Soft tissue sarcomas include non-epithelial tumours, excluding tumours of the haematopoietic system, lymph nodes, skeleton and central nervous system. However, tumours of the peripheral nerve system are classified as soft tissue tumours. Contrary to other customary classifications, also non-epithelial tumours of the internal organs such as the stomach and uterus are discussed in this consensus text, since their diagnostics and treatment do not virtually differ from those occurring in other sites. Paediatric tumours (age $< 16$) are disregarded because of their specific problems.

### *2. Classification of soft tissue sarcomas according to Enzinger and Weiss[1]' is currently most common*

Prognosis of soft tissue sarcomas is determined by grade of malignancy and histological typing. Over the years many classifications have been proposed. Currently, the classification according to Enzinger and Weiss is used most, comprising in short:

fibrosarcoma dermatofibrosarcoma protuberans

[1]*Soft Tissue Tumors*, 2nd edition. St. Louis: C. V. Mosby, 1983, p. 13.

Correspondence to: Irma P. M. van Beuningen, Secretary NWWDT, p/o IKMN, Servaesbolwerk 14, 3512 NK Utrecht, The Netherlands. Tel: + 31.30.2314868; Fax: + 31.30.2316587.

1357-714X/98/030183-09 $9.00 © 1998 Carfax Publishing Ltd malignant fibrous histiocytoma
liposarcoma
- well differentiated
- mixoid
- round cell
- pleomorphic leiomyosarcoma
rhabdomyosarcoma
rhabdoid sarcoma
angiosarcoma
malignant haemangiopericytoma
synovial sarcoma
malignant Schwannoma
peripheral primitive neurectodermal tumour
extraskeletal chondrosarcoma
- myxoid
- mesenchymal extraskeletal osteosarcoma
malignant granular cell tumour
alveolar soft tissue sarcoma
epitheloid cell sarcoma
clear cell sarcoma
extraskeletal Ewing's sarcoma

It is not always possible to classify a sarcoma into one of the above types. Such tumours are classified as 'not otherwise specified sarcoma' (NOSS). This classification does not include malignant mesothelioma because of its typical localization and corresponding clinical behaviour, or locally aggressive non-metastasizing tumours such as (aggressive) fibromatosis.

Soft tissue tumours can principally occur in all sites of the body. Most frequent localizations are:

| | | |
|---|---|---|
| lower extremity | 40% | (of which 75% above the knee) |
| upper extremity | 15% | |
| chest/abdominal wall | 15% | |
| head and neck | 15% | |
| retroperitoneum | 10% | |

### Epidemiology

*3. Soft tissue sarcomas are uncommon and their frequency in south-east Holland has not significantly increased since 1975*

According to the National Cancer Registration, soft tissue sarcomas comprised $<1\%$ of all new malignancies in The Netherlands in 1989 and 1990. In a total of 55,000 new cancer patients per year, 691 patients were diagnosed with sarcoma, of whom 364 had a soft tissue sarcoma. Incidence rates were 2.7 in males and 2.1 in females per 100,000 per year. According to a special documentation by the IKMN (Comprehensive Cancer Centre for central Holland) cancer registration, apart from these 364 patients, another 90 patients (25%) had a low malignant tumour included in the policy discussed here.

The long-term cancer registration by the IKZ (Comprehensive Cancer Centre for South Holland) in Eindhoven shows no increase in the incidence of soft tissue sarcomas; however, it does show a significant increase in the number of small tumours and a decrease in the percentage of larger tumours of unknown size.

### Diagnostics

*4. Recognize an uncommon tumour!*

In a patient suspected of having a soft tissue tumour, a sarcoma should be assumed until proof to the contrary; assessment of complaints and growth speed and of local tumour growth, and regional and distant metastases is necessary, including accurate assessment of tumour size (in cm) and involvement of surrounding structures. A fast-growing tumour (i.e. in some weeks to months) or a tumour situated under the deep fascia should suggest a strong suspicion of a malignancy. A solid consistency and an unusual localization should also alarm the clinician.

In case of the slightest suspicion of a malignancy, surgical exploration of a soft tissue tumour should adhere to a number of rules. The prognosis of soft tissue sarcomas at presentation depends on two factors only: local tumour extension (local and/or distant metastasis), and the biological behaviour of the tumour, expressed as 'grade of malignancy'. The latter prognostic factor cannot be influenced; however, previous inexperienced manipulation can indeed unfavourably influence the first, hence complicating definitive treatment. Contamination of nearby muscle groups or compartments by invasive examination necessitates involvement in the definitive treatment. Therefore, it is highly recommended that diagnostic imaging (for localization and staging) is performed prior to invasive diagnostics and particularly prior to biopsy.

*5. Diagnostic imaging before every invasive examination*

Imaging techniques that can be used are: conventional X-rays, ultrasound (us), MRI and/or CT. Bone scintigraphy and arteriography are not indicated, except in special situations that cannot be included in a protocol. Only in exceptional cases can a probability diagnosis based on diagnostic imaging be made. Usually diagnostics start with conventional X-rays that can give an impression of the extension of the tumour and of skeletal involvement. It can often differentiate the diagnosis of a primary soft tissue process between a primary osteosarcoma or a soft tissue tumour with skeletal infiltration (e.g. in synovial sarcoma). Vascular calcifications may indicate haemangioma. Ultrasound is a low-cost, fast and non-invasive method to get a first impression of presence, localization and extension of a clinically diagnosed tumour. Sometimes the nature of the tumour can be further defined.

*6. MRI is the most reliable method to assess localization and size of a soft tissue tumour, as well as involvement in several anatomical compartments*

Magnetic resonance imaging (MRI) is a most reliable method to define exactly the localization and macroscopic extension of the tumour. Not only tumour size, but also involvement in several anatomical compartments (muscle groups, cortex, bone marrow, neurovascular bundle, joint) can be accurately assessed. It is possible to identify the tumour, the pseudocapsule and the reactive zone. Since tru-cut and surgical (open) biopsies cause reactive changes that are difficult to differentiate from tumour tissue by MRI. MRI should be performed prior to these invasive procedures. At least two T1 and T2 weighed perpendicular MRIs (e.g. in the transversal and sagittal directions) should be made.

CT is less reliable than MRI. CT is preferred in instances of an intra-abdominal tumour, such as leiomyosarcoma of the stomach. In tumours originating from muscle and connective tissue, etc. CT is only indicated when MRI cannot be realized because of compelling practical or patient-related reasons (claustrophobia, pacemaker, eye lens implant).

In a number of cases, particularly benign diseases, a specific diagnosis can be made by MRI.

*7. Since soft tissue sarcoma in general metastasizes primarily to the lung, screening for lung metastases is a routine procedure in all patients with a soft tissue sarcoma*

For lung screening, conventional X-rays and CT-thorax are used. Timing of X-thorax is not critical. It can be performed at the start of analysis (in case of a strong clinical suspicion), or alternatively after histologically diagnosing a soft tissue tumour. CT is indicated when histology shows a malignant tumour, whereas X-thorax does not indicate the presence of lung metastases.

*8. Cytology is valuable in assessing a recurrent soft tissue tumour*

Cytology is not the first choice to diagnose a soft tissue tumour. When differentiation of a primary tumour between a soft tissue tumour, an epithelial tumour, a melanoma or a lymphoma is sufficient, cytology can generally be conclusive. In tumours surgically difficult to access, an ultrasound-guided cytological biopsy can be taken. The biopsy site should be marked. Cytological examination by a fine needle biopsy can be useful to assess recurrence or metastasis of a known sarcoma. A tru-cut biopsy is indicated in tumours for which a cytological biopsy is inconclusive. For tissue obtained by tru-cut biopsy only a limited histopathological classification and/or staging can be assessed. By performing a tru-cut biopsy, a tissue cylinder is obtained directly from the tumour periphery, hence avoiding non-involved muscle groups. Specimens obtained from the tumour centre often yield necrosis. The biopsy site is sutured; the suture remains *in situ* until definitive treatment.

*9. Also an incisional biopsy is aimed at a combined treatment of surgery and radiotherapy without unnecessary mutilation*

Except in small superficial tumours the aim of surgical exploration should be kept in mind: to obtain histopathological material and *not* to be a definitive treatment for soft tissue sarcoma.

In performing an incisional biopsy, the following guidelines should be followed:

- avoid contamination of other muscles/muscle groups or compartments as a result of infiltration anaesthesia, tumour manipulation, haematoma and infection;
- perform the biopsy by a longitudinal incision;
- do not undermine skin margins;
- take the biopsy from the tumour periphery (including the pseudo-capsule);
- wash the wound with cytolytic fluid (sterile water, Dakin-fluid);
- perform accurate haemostasis;
- when using a drain, do not release this through a separate drain hole;
- use sterile instruments when closing the wound;
- send fresh specimens to pathology (eventually frozen sections, re: 'representative material?').

*10. Send fresh soft tissue tumour specimens to pathology, indicating margins, growth speed and previous treatment*

In requesting a pathology review, the following should be specifically recorded:

- tumour localization (superficial or deep) and in case of a biopsy, biopsy specimen or partial resection, and tumour sizes;
- recurrence: yes or no;
- previous treatment (if applicable).

The resected tumour should be intact and fresh when handed to the pathologist. Surgical adequacy can then be judged best and before total fixation tumour fragments can be removed for freezing purposes and separate fixation for EM and other tests.

*11. Pathology review is aimed at assessing a definitive, classifying diagnosis and at determining the extension of the soft tissue tumour, the extent of surgical adequacy in case of a malignancy, and prognostic factors influencing treatment*

Pathology reviews and reports should include the following:

- surgical nature (incisional biopsy, excisional biopsy, radical resection, etc.);
- tumour sizes, preferably in three dimensions, but minimally mentioning the largest diameter;
- surrounding tissues and involvement with the tumour (infiltration: yes or no): skin, fascia, muscle, fatty tissue, nerve, bone;
- relation between the tumour and the resection lines, particularly the possible presence of macroscopic disease in this area. Marks applied by the surgeon to reconstruct the original position of the tumour in the body should be used here. Resection lines should be marked, e.g. with Indian ink;
- thickness of the cuff of normal tissue surrounding the tumour, with special attention for those sites indicated as non-radical resections (because of technical difficulty). Muscle retraction can hinder judgement of treatment adequacy. It is very useful and sometimes necessary that the pathologist examines the macroscopic specimen together with the surgeon. The conclusions of this joint examination should be recorded in writing (schematic drawing!);
- tumour consistency and shape (clear margins: yes or no; nodular), as well as its aspect in diameter, especially colour, and presence and extension of myxoid parts and necrosis;
- non-radical resections based on macroscopic observations and the marks applied by the surgeon. Fractions of these areas should be prepared as well and should be filed such, preferably based on a schematic drawing, that their origin can be reconstructed. For microscopic diagnosis of the nature of the tumour a sufficient number of tissue specimens should be removed as well. As a guideline two fractions will be sufficient for a tumour with maximum diameter up to 5 cm; a tumour > 5 cm will need the number of fractions corresponding to half of the largest diameter in centimetres. It is not particularly useful to prepare fractions from completely necrotic areas. However, necrosis should be microscopically assessed. Particularly, fractions should be prepared from—macroscopically described—areas with a deviating aspect;
- grading;
- the conclusion should minimally include the following:
  - (i) surgical nature
  - (ii) localization
  - (iii) previous treatment (if applicable)
  - (iv) histological typing and grade
  - (v) tumour size(s)
  - (vi) data on treatment adequacy

*12. Classification in grades of malignancy and classifying diagnosis are equally important for soft tissue sarcoma prognosis*

To define prognostic correlations, grading and histological typing of soft tissue sarcoma are equally important. Most publications appear to acknowledge prognostic value to histological typing, but in general this value disappears when in a multivariate analysis histological typing is tested as a variable next to grading.

In this context it is striking that a generally accepted system exists for histologically typing these sarcomas, but not (as yet) for grading these malignancies. From recent analysis it appears time and again that by multivariate analysis the number of mitoses, and the presence and amount of necrosis are decisive for prognosis. This also appears from a recently published EORTC study on which the following classification is based:

- grade 1: $<4$ mitoses per 2 $mm^2$
- grade 2: 4–25 mitoses per 2 $mm^2$
- grade 3: $>25$ mitoses per 2 $mm^2$

In tumours that can locally be treated adequately, a 5-year survival can be expected for over 90% of grade 1 tumours, $\pm 70\%$ for grade 2 tumours and $\pm 45\%$ for grade 3 tumours. We may assume that this system includes malignant fibrous histiocytoma (MFH), liposarcoma, leiomyosarcoma, fibrosarcoma, malignant Schwannoma, and synovial sarcoma, together comprising $>86\%$ of diagnosed sarcomas. In other sarcomas the grade of malignancy should be estimated based on histological typing. This grading system excludes sarcomas of the internal organs. In the study on which this system is based, the presence or absence of necrosis merely renders the possibility to divide grade/score 3 tumours into two additional grades. In practice, no need appears to do this.

NB: the number of mitoses cannot simply be used to differentiate between a benign (i.e. reactive) and a malignant mesenchymal proliferation. Benign mesenchymal proliferations exist with a large number of mitoses.

*13. Aggressive fibromatosis and similar tumours are treated as locally malignant*

Apart from sarcomas and benign tumours with only limited growth potential, a group of soft tissue tumours can be distinguished with unlimited growth potential, but without a risk for metastasis. These tumours can be classified as 'locally malignant'. Recurrence is due after inadequate local treatment. Since surgical treatment of a recurrence generally is more extensive and more mutilating, adequate local treatment is indicated first.

For another group of tumours, histopathology cannot predict their clinical behaviour (such as probability of recurrence). This group is termed 'tumours of intermediate malignancy' (according to Enzinger and Weiss). In these tumours the extent of surgery depends on considering both the disadvantages of locally adequate treatment and the possibility of adequately treating a recurrence.

### Treatment

*14. Soft tissue sarcoma requires multidisciplinary treatment from the start. The team are acquainted with each other's diagnostic and therapeutic possibilities*

Surgery is the first line of treatment for soft tissue tumours. Conditions for proper surgical treatment are:

- pre-operative staging (cTNM);
- pre-operative review with the consultant representing the hospital's Oncology Working Party (Comprehensive Cancer Centre);
- preferably pre-operative assessment by a radiation oncologist;
- preferably one adequate operation;
- marking of areas at risk and narrow margins with titanium staples.

*15. Direct primary excision only indicated for small superficial soft tissue tumours*

Primary resection (excisional biopsy) should only be considered for very small ($<3$ cm) superficial tumours, for which surgery will not render functional loss. As in resection, also for these primary resections, wide margins are required.

Radical resection is resection of the tumour *en bloc* including a cuff of normal tissue of at least 2 cm. For tumours infiltrating muscular compartments compartmental resection may be considered.

If radical resection implies sacrificing the neurovascular bundle or bone, causing severe morbidity, a combined treatment of surgery and radiotherapy is preferred.

For retroperitoneal or head and neck lesions, radical resection will often be impossible for anatomical reasons. A less radical resection and post-operative radiation will be the treatment of choice.

*16. Surgical reports should completely describe all conclusions, anatomical structures and areas at risk*

The surgical report should include the following:

- operation, approach and vital structures;
- a schematic drawing of the resection, particularly indicating margins and anatomical structures;
- areas at risk (narrow margins);
- haemoclips used as landmarks;
- size of the tumour in centimetres;
- reconstruction.

*17. Inadequate surgery is preferably followed by reoperation, after consulting the Oncology Group*

In case of inadequate resection, when clearance margins show evidence of residual macroscopic tumour (according to the surgical report/pathology conclusions), re-resection of the entire contaminated area should be performed if possible. If re-resection is not possible, radiotherapy should be applied.

*18. Retroperitoneal sarcoma should be treated by a properly prepared radical resection, if possible followed by radiation to the tumour bed*

Because of anatomical reasons, radical resection sometimes implies resection of surrounding structures as well, such as the colon, ureter, iliacal vessels, etc., with optional reconstruction. Pre-operative review with the specialists in charge is strongly recommended. Margins often appear to be inadequate still. Therefore, treatment principally requires a combined planning of surgery and radiotherapy.

*19. After surgical treatment of retroperitoneal sarcoma the use of the omentum or a spacer should be considered to prevent radiation damage*

To apply a sufficient radiation dose to the original tumour volume, precautions should be taken to prevent irreversible small bowel radiation damage, a long-term complication. One of these precautions is retraction of the small bowel from the surgical area.

*20. Radiotherapy is indicated after planned non-radical resection, when the resection margin is $<1$ cm after fixation, or after incomplete resection of the original surgical area*

The development of megavoltage radiation equipment and modern radiation techniques enables accurate radiation, hence sparing surrounding normal tissues. In patients with a limited tumour process, post-operative adjuvant radiotherapy appears to reduce the risk of a local recurrence.

In this combined treatment radiotherapy can be performed pre-operatively or post-operatively. Post-operative radiotherapy has been applied most. The advantages of post-operative radiation over pre-operative radiation are:

- no delay of surgery;
- no increased risk of post-operative complications;
- optimal information on the extent, margins and histological aspects of the tumour, to determine the treatment volume;
- the radiation oncologist can inform himself of the exact tumour extent during surgery.

Post-operative radiotherapy is indicated for all G3 tumours and recurrences, even after radical surgery. In all other cases post-operative radiotherapy is indicated by the result of surgery:

- after non-radical surgery, when reoperation is impossible or too mutilating;
- after reoperation because of non-radical surgery, when the entire surgical area has not been removed;
- after marginal radical surgery (margin $<2$ cm [fresh specimen] or $<1$ cm [after fixation]);
- in case of tumour contamination ('spill') during surgery.

The target area for post-operative radiotherapy includes the entire original tumour volume, possible microscopic extension and areas possibly contaminated during resection (drain, haematoma). To determine the target area a complete description of the surgical procedure and the pathology report, as well as pre-operative CT and/or MRI scans should be available to the radiation oncologist. Radiation margins depend on tumour localization and extent.

Guidelines for determining the target area are:

- In intracompartmental tumours, the related compartment is considered the target area.
- Margins for extracompartmental high-grade tumours (grades 2 and 3) are 7–10 cm longitudinally from the original tumour, taking natural margins into account. For low-grade tumours the margin can be 5 cm. Margins should be wide, particularly in the direction of vessels and nerves and of fascia and muscle fibers. Transversely, a margin of at least 2 cm can be chosen, again taking into account natural margins of anatomical structures.
- In subcutaneous tumours not infiltrating the fascia and not situated near vessels and nerves, a margin of 5 cm around the original tumour will be sufficient.
- For the booster dose the target area is the original tumour bed with a margin of 2 cm. After a non-radical resection the highest dose is applied to an area that is preferably marked during surgery. The margin is determined by the treatment volume, the total dose, and normal tissue tolerance.

Radiation is generally performed using multiple megavoltage photon beams. A CT-scan in radiation position can be used to determine the treatment plan and to calculate the dose distribution. In case of radiotherapy of an extremity, beam directions should be chosen sparing non-affected muscle compartments and bone as much as possible. The interosseous membrane of the distal limb can serve as the margin between the irradiated and the non-irradiated part of the extremity. Radiotherapy to the entire limb periphery should be avoided as much as possible to preserve lymph drainage. Haemoclips placed in the bottom and at the margins of the surgical area can be useful to determine the treatment volume. Patient positioning reproducibility may benefit from proper patient fixation in radiation position. To this end, several methods are available. Radiotherapy to retroperitoneal or abdominal sites may be difficult due to surrounding radiosensitive organs such as kidneys and bowel. Placing a spacer (either a tissue expander filled with saline or a silicone breast prosthesis) or a pedicled omentoplasty between the treatment volume and these organs enables application of a higher radiation dosage.

ICRU dose recommendations are 50 Gy in 25 fractions in 5 weeks to the above treatment volume, plus 10 Gy in 5 fractions applied similarly to the original tumour volume, including the above-mentioned margins.

*21. Post-operative interstitial radiotherapy enables more accurate determination of the booster area and sparing of healthy tissues*

When surgery is performed in a hospital with brachytherapy facilities, the radiation oncologist can, immediately following excision, insert loops in the original tumour area for post-operative interstitial application of an iridium booster dose using an afterloading system. This approach requires intensive consultation between the surgeon and the radiation oncologist before the start of treatment. Preferably, the patient is also assessed by the radiation oncologist before treatment. Technique and dose of interstitial radiotherapy are presently studied.

*22. During radiotherapy to an extremity and at least during 1 year thereafter physiotherapy is prescribed to prevent contractures*

After high-dose radiotherapy some fibrosis and skin discolouration is often seen. Severe complications after radiotherapy may be: function impairment due to fibrosis of muscles and subcutaneous tissues; joint ankylosis; lymph oedema; and vascular insufficiency due to vascular damage. Also bone fractures due to irradiation are seen until long after treatment.

Accurate assessment of the radiation technique with sparing of as much healthy tissue as possible decreases the risk of these complications. After a combined treatment of surgery and radiotherapy, long-term physiotherapy is highly important to preserve extremity function. These exercises, aimed at keeping muscles and tendons at full length, should be done under the guidance of a physiotherapist and preferably after consulting a rehabilitation physician. They should be started prior to and during radiotherapy and be maintained for 1–2 years after treatment.

*23 Chemotherapy for primary and metastasized soft tissue sarcoma is experimentally administered in trials*

Only three cytostatic drugs have proved to be effective in soft tissue sarcoma (adriamycin, ifosfamide and, to a lesser extent, DTIC). Dose–effect relations, combined treatment schedules and the addition of growth factors are currently under investigation. Systemic therapy is applied in trials only. Adjuvant chemotherapy in high-risk patients is not indicated as yet. The value of chemotherapy prior to radical surgery has not been assessed yet, nor when administered intra-arterially in extremity lesions. Induction (neo-adjuvant) chemotherapy is administered in trials only.

*24. Prior to resection of lung metastases a number of terms have to be met: (i) the primary tumour should be under control; (ii) exclusion of extrapulmonary metastases; (iii) no contraindications for pulmonary surgery*

Soft tissue sarcoma usually metastasizes primarily to the lungs. Therefore, all patients with soft tissue sarcoma should be examined for lung metastases. Since diagnosing lung metastases is clinically relevant, chest X-ray is performed first. A negative chest X-ray does not rule out lung metastases and a CT-thorax should follow. Radical surgery of lung metastases can accomplish cure or long-term complete remission. Lung function is extremely important in patients who had previous chemotherapy. There should be no contraindications for pulmonary surgery.

Adequate patient selection can render a 5-year survival of 30–40%. One factor is of utmost importance: radical metastectomy should be feasible. Prognosis after radical metastectomy in grade 3 tumours seems less favourable than in grade 1 and grade 2 tumours.

The choice between a sternotomy and a unilateral or bilateral thoracotomy strongly depends on localization and the number of metastases.

*25. Embryonal soft tissue sarcomas in adults are treated as paediatric tumours*

Although the available separate studies do not show this numerically, analysis of a large number of publications using chemotherapy (doxorubicin + DTIC, or this combination plus supplementary endoxan and vincristin) shows no difference in response to chemotherapy in several histological types of soft tissue sarcoma. Embryonal rhabdomyosarcoma in adults is an exception. The clinical behaviour of this subtype is somewhat different: rather fast-growing, extensive haematogenic metastasis and a striking sensitivity to chemotherapy. Whereas chemotherapy for other histological subtypes renders response rates of 20–40%, the response rates of embryonal rhabdomyosarcoma to chemotherapy is 80–90%. In general, drugs such as vincristine and actinomycin have little effect on soft tissue sarcoma. However, these drugs do appear to be effective in embryonal rhabdomyosarcoma. Based on these data, it is recommended to treat embryonal rhabdomyosarcoma analogous to paediatric tumours with chemotherapy, such as Ewing's sarcoma and nephroblastoma.

*26. If resection of a soft tissue sarcoma of the limb leads to amputation or severe morbidity, an isolated regional perfusion using TNF-α should be considered*

Regional isolated perfusion has been used as an alternative to amputation. This method enables regional administration of high doses of chemotherapy in soft tissue sarcoma of the limbs without the risk of (lethal) toxicity. However, despite the use of different regimes the results are disappointing regarding the choice of cytostatics as well as the application of hyperthermia.

Tumour Necrosis Factor-α (TNF-α) is a cytokine with an acute and dramatic anti-tumour effect in animal tumour models. Effective doses in animals are many times ($>20\times$) higher than the maximum allowed dose in humans. Systemic administration of TNF-α in humans causes severe toxicity without reaching a significant anti-tumour effect. Effective TNF-α levels can be reached in isolated regional perfusion of the limbs.

The anti-tumour effect of TNF-α is probably based predominantly on selective total destruction of tumour vessels. This is illustrated by pre- and post-perfusion angiographies, as well as by histopathological findings. Melfalan has a strongly synergistic effect in combination with TNF-α. The tumours usually react with acute softening, as an expression of massive necrosis, followed by tumour regression, hence turning the tumour mobile and resectable.

In a multi-centre TNF study in Rotterdam (DDHCC), Groningen (University Hospital), Amsterdam (Antoni van Leeuwenhoek Hospital) and Lausanne (Centre Pluriforme d'Oncologie) patients with an irresectable soft tissue sarcoma of the limb are treated.

An anatomical or functional amputation can be prevented in ±90% of the patients by performing an isolated perfusion, followed by a generally minor resection of tumour remnants, instead of amputation or a mutilating combination of major resection and radiotherapy.

Response percentages of soft tissue tumours to TNF perfusion are impressive: CR 44%, PR 53% and SD 3%. However, it has not (yet) rendered a benefit in survival.

*27. All patients with soft tissue tumours are preferably treated in trials*

Soft tissue tumours are uncommon tumours with a large scale in histology and grading. Adequate local treatment reduces the risk of local recurrence to 10–20%; however, 40% of patients with soft tissue tumours will develop distant metastases.

In order to get more insight in all tumours and to improve the prognosis of patients with soft tissue tumours, it is essential to participate in trials.

*28. Rarity and diversity of soft tissue sarcoma and the uncertainties and complexity of the treatment argue for a national approach in documenting diagnostics, staging and treatment*

Soft tissue sarcoma rarity is shown by the fact that the average surgeon and a pathologist see a few new cases only once every 2 years and once a year, respectively. Moreover, there is a diversity in localizations (for the surgeon) and in histological typing (for the pathologist).

National documentation in this field gives a unique opportunity to acquire a better insight into the behaviour, diagnostics and treatment of these tumours. Large-scale studies have until now exclusively been performed in centres meeting a selection of patients. It is of utmost importance to start a larger databank than the current one used for cancer registration. Before filing the data, the main questions related to these tumours should be considered.

### Appendix 1

*Follow-up*

Follow-up should be aimed at:

1. control of (functional) recovery after treatment
2. recognition of a local recurrence
3. recognition of (a) lung metastasis (metastases)
4. recognition of late complications

*Frequency of follow-up*

| | |
|---|---|
| 1st and 2nd year: | every 3 months |
| 3rd, 4th and 5th year: | every 6 months |
| >5 years: | once per year |
| After 10-year disease-free survival: | stop follow-up |

*Contents of follow-up*

| | |
|---|---|
| Always: | history—general and specific physical examination |
| Every 6 months during the first 2 years: | CXR/yearly thereafter |
| When indicated: | CT-scan or MRI |

Indications for CT or MRI may be:

- determining post-operative starting point in tumour areas difficult to examine, e.g.
  - (i) sarcomas of the head and neck
  - (ii) retroperitoneal sarcomas
  - (iii) sarcomas of pelvis and hip
- follow-up of local control after doubtful radical surgery
- in case of complaints or clinical suspicion.

### Appendix 2

*TNM classification (UICC 1992, adults)*

The TNM (Tumour, Node, Metastasis) classification is exclusively used in adults (minimal age 16 years) and for histologically proven sarcomas.

Minimally required procedures for assessing the *T category*: physical examination and imaging (MRI or CT-scan and/or ultrasound) of the primary tumour:

*T* = *Primary tumour*

Tx = Primary tumour cannot be assessed

T0 = No evidence of primary tumour

T1 = Tumour diameter 5 cm or less

T2 = Tumour diameter >5 cm

Minimally required procedures for assessing the *N category*: physical examination:

*N* = *Regional lymph nodes*

Nx = Regional lymph nodes cannot be assessed

N0 = No regional lymph node metastasis

N1 = Regional lymph node metastasis

Minimally required procedures for assessing the *M category*: physical examination, laboratory examinations and chest X-ray (if necessary, CT-thorax):

*M* = *Distant metastasis*

### References

Brant TA, Parsons JT, Marcus RB, *et al.* Irradiation *for* soft tissue sarcomas of the trunk and extremities in adults. *Int J Radiat Oncol Biol Phys* 1990; 19:899–906.

Carson AG, Putman JB, Natarjan G, *et al.* Five year survival after pulmonary metastasectomy for adult soft tissue sarcoma. *Cancer* 1992; 69:662–8.

Cionini L, Marzano S, Olmi P. Soft tissue sarcomas: experience with intraoperative brachytherapy in the conservative management. *Ann Oncol* 1992; 3 (Suppl. 2):S63–S66.

Collin C, Godbold J, Brennan M. Localized extremity soft tissue sarcoma: an analysis of factors affecting survival. *J Clin Oncol* 1987; 5:601–12.

Elias AD, Curtman H. Adjuvant chemotherapy for soft tissue sarcoma: an approach in search of an effective regime. *Scm Oncol* 1989; 16:305–11.

Enzinger FM, Weiss SW. Soft tissue tumours. St. Louis [etc.]: Mosby 1988. Guiliano AE, Eilber FR. The rationale for planned re-operation after unplanned total excision of soft tissue sarcomas. *J Clin Oncol* 1985; 3:1344–8.

Habrand JL, Gerbaulet A, Pejovic MH, Contesso G, Durand S, Haie C, *et al.* Twenty years experience of interstitial iridium brachytherapy in the management of soft tissue sarcomas. *Int J Radiat Oncol Biol Phys* 1991; 20:405–11.

Kransdorf MJ. Jelinek IS, Moser RP. Imaging of soft tissue tumors. *Radiologic Clin N Am* 1993; 31:359–72.

Lienard D, Lejeune FJ, Delmotte JJ, Renard N, Ewalenko D. High doses of rTNF-$\alpha$ in combination with IFN-gamma and melphalan in isolated perfusion of the limbs for melanoma and sarcoma. *J Clin Oncol* 1992; 10:52–60.

Markhede G, Angervall L, Stener B. A multivariate analysis of the prognosis after surgical treatment of malignant soft tissue tumours. *Cancer* 1982; 49:1721–33.

Martini N, McCormack PM. Pulmonary resection in sarcoma metastases. In: Ryan JR ed.: *Recent concepts in sarcoma treatment*. Dordrecht: Kluwer, 1988; 197–200.

Mills EED, Hering ER. Management of soft tissue tumours by limited surgery combined with tumour bed irradiation using brachytherapy and supplementary teletherapy. *B J Radiol* 1981; 54:312–7.

Mankin HJ, Lange TA. Spanier SS. The hazards of biopsy in patients with malignant primary bone and soft tissue tumors. *J Bone Joint Surg* 1982; 64A:1121–7.

Russell WO, Cohen J, Enzinger FM. *et al.* A clinical and pathological staging system for soft tissue sarcoma. *Cancer* 1977; 40:1562.

Rydholm A. Berg NO, Persson BM *et al.* Treatment of soft tissue sarcoma should be centralized. *Acta Orthop Scand* 1983; 54:333–9.

Shiu MH, Brennan MF. *Surgical management of soft tissue sarcoma*. Philadelphia/London: Lea and Febiger, 1989.

Shiu MH, Hilaris BS, Harrison LB, Brennan MF. Brachytherapy and function-saving resection of soft tissue sarcoma arising in the limb. *Int J Radiat Oncol Biol Phys* 1991; 21:1485–92.

Steward WP, Verwey I, Somers R, *et al.* Granlocyte macrophage colony-stimulating factor allows safe escala-tion of dose-intensity of chemotherapy in metastatic adult soft tissue sarcomas: a study of the EORTC soft tissue and bone sarcoma group. *J Clin Oncol* 1993; 11:15–21.

Stinson SF, DeLaney TF, Greenberg J, *et al.* Acute and long-term effects on limb function of combined modality limbsparing therapy for extremity soft tissue sarcoma. *Int J Radiat Oncol Biol Phys* 1991; 21:1493–9.

Suit HD, Mankin HJ, Wood WC, *et al.* Preoperative, intraoperative and postoperative radiation in the treatment of soft tissue sarcoma. *Cancer* 1985; 55:2659–67.

Tepper J, Rosenberg SA, Glatstein E. Radiation therapy technique in soft tissue sarcomas of the extremity—policies of treatment at the National Cancer Institute. *Int J Radiat Oncol Biol Phys* 1982; 8:263–73.

Unnik JAM van, Coindre JM, Contesso G, Albus-Lutter ChE, Schiodt T, Garcia-Miralles T, *et al.* A Grading of soft tissue sarcomas: experience of the EORTC soft tissue and bone sarcoma group. *Euro J Cancer* 1993; 29A:2089–93.
